# AlScN Piezoelectric MEMS Mirrors with Large Field of View for LiDAR Application

**DOI:** 10.3390/mi13091550

**Published:** 2022-09-18

**Authors:** Yichen Liu, Lihao Wang, Yongquan Su, Yuyao Zhang, Yang Wang, Zhenyu Wu

**Affiliations:** 1 State Key Laboratory of Transducer Technology, Shanghai Institute of Microsystem and Information Technology, Chinese Academy of Sciences, Shanghai 200050, China; 2 School of Microelectronics, Shanghai University, Shanghai 200444, China; 3 Shanghai Industrial Technology Research Institute, Shanghai 201800, China; 4 University of Chinese Academy of Sciences, Beijing 100049, China

**Keywords:** MEMS mirror, piezoelectric, AlScN, mechanical coupling, Lissajous scanning, LiDAR

## Abstract

This paper presents AlScN piezoelectric two-axis MEMS mirrors with gimbal-less and gimbaled designs fabricated in a CMOS-compatible manner. Integrated piezoelectric sensors provided feedback signals of the actual mirror positions. The mirror with a diameter of 1.5 mm possessed adjustable optical tilt angles of up to 22.6° @ 30 V, with a high resonance frequency of about 8.2 kHz, while the 3 mm mirror reached 48.5° @ 41 V. The mirror with the gimbaled structure exhibited an excellent field of view and good mechanical decoupling. Additionally, a significant improvement in mirror scanning performance was observed in a vacuum (4 Pa), proving that the optical field of view was magnified by more than a factor of 10.

## 1. Introduction

LiDAR (Light Detection and Ranging) has shown its importance in many areas, such as topography [1], 3D imaging [2,3,4,5,6], spectroscopy [7], surgery [8], and automobile engineering [9,10,11]. With the emergence of new fields, such as autonomous driving and ADAS (Advanced Driver Assistance System), the technical demand for surrounding environment perception is rapidly increasing. LiDAR is increasingly used in related fields thanks to its advantage of building a 3D point cloud, where the light scanning device is one of the core components. The MEMS mirror is one of the emerging scanning devices due to its fast scanning speed, compact structure, suitability for mass production, and high-quality point cloud output [12,13]. However, recent MEMS LiDAR systems have drawbacks, such as a short detection range and a relatively small field of view (FOV). Hence, there is demand for the design of micromirrors with a large aperture and a large tilt angle.

There are four main types of driving mechanisms for MEMS mirrors. Electrostatic driving mechanisms are usually hindered by their inherent nonlinear electrostatic force, which leads to control complexity [14]. Electromagnetic driving mechanisms have good linearity, but the nonnegligible energy consumption is still a demerit [8]. The working frequency of mirrors driven by an electrothermal actuator is usually limited because of the slow response time [2]. By contrast, piezoelectric driving mechanisms possess high linearity, low power consumption, and high scanning speeds, and their use is consequently a suitable actuation method for MEMS mirrors [15].

Lead zirconate titanate (PZT) films, which possess high piezoelectric coefficients, are widely utilized as functional layers in many kinds of piezoelectric MEMS mirrors [16,17,18,19,20,21,22]. In recent studies, AlN and Sc-doped AlN (AlScN) films are becoming more and more popular on account of their hysteresis-free characteristics. In 2018, Shao et al. reported a scanning AlN-based piezoelectric micromirror in which a tilt angle of 1° @ 5 V was achieved with a 200 μm × 200 μm mirror plate. [23] Lei et al. developed a single-axis MEMS mirror with a larger mirror plate (6 mm × 4 mm) which reached an optical angle of 8.2° @ 10 V [24]. In another circular scanning mirror, described in 2019 by Pensala et al., the maximum optical angle achieved was 30° @ 2 V with a vacuum package [11]. Gu-Stoppel et al. carried out work in 2020 on MEMS mirrors based on AlN and reached a tilting angle of 55° @ 80 V in a vacuum [25]. The tilting angles reported in these articles were limited by the piezoelectric coefficient of AlN and were always compensated by a vacuum package. Therefore, a piezoelectric material with a higher piezoelectric coefficient than AlN is desired. In this regard, AlScN is a suitable material for piezoelectric driving because of its high linearity and long-term stability compared to PZT, as well as a higher piezoelectric coefficient compared to undoped AlN. Additionally, the high voltage tolerability of AlScN compensates for its piezoelectric coefficient deficiencies. In 2021, Gu-Stoppel et al. developed a quasi-static MEMS mirror using AlScN that realized a static mechanical tilting angle of 8° @ 150 V according to the simulation result, showing that AlScN is an ideal material for piezoelectric-driven MEMS micromirrors.

In this paper, AlScN film-based piezoelectric MEMS mirrors are presented. The designed mirrors all have two resonant axes for Lissajous scanning, to meet the requirements for LiDAR application. The mirrors utilize AlScN film as the functional layer, which has a higher piezoelectric coefficient, and so enables a more acceptable optical tilting angle to be achieved in air conditions. Further, the scanning performance in a vacuum is also implemented to reveal how the results in this case differ from the results obtained in air.

In this article, the design parameters and simulation results of gimbal-less and gimbaled micromirrors are illustrated in Section 2. Section 3 describes the MEMS fabrication process. Then, the characterization and scanning performance of the mirrors are analyzed in Section 4. Finally, a discussion of the proposed mirrors and conclusions regarding them is presented in Section 5.

## 2. Principle and Design

In order to produce a 2D scanning area with MEMS mirrors for LiDAR application, the laser needs to be deflected in two directions at the same time. Therefore, a two-axis MEMS mirror is desired. The 2D scanning mirrors that have been described in previous publications can be divided into two types: those with central symmetrical actuators (known as ‘gimbal-less mirrors’) [22,25,26,27,28], and those with individual axes resonating orthogonally (known as ‘gimbaled mirrors’) [16,29,30,31]. Generally, gimbal-less mirrors possess a high resonant frequency for both axes and have the advantage of a high fill factor. However, the mechanical coupling of the two axes is difficult to eliminate completely; whereas a gimbaled mirror structure allows for a physical decoupling of the two axes but it is limited by a relatively poor fill factor and a lower slow axis scanning speed. With the purpose of studying the pros and cons of these two types of mirrors, this paper presents both kinds of piezoelectrical designs for comparison.

### 2.1. Mirror with Gimbal-Less Structure (Design I)

**Figure 1:**
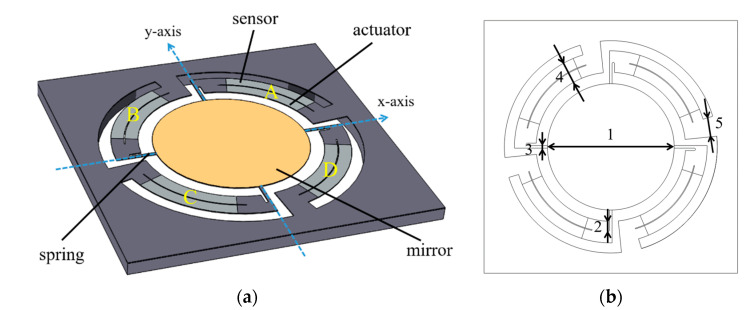
(**a**) The structure of *Design I*. (**b**) *Design I* parameter index.

The structure of *Design I* can be found in Figure 1. Four identical cantilevers anchored at the Si substrate surround the mirror and connect to it with springs. The silicon and piezoelectrical device layers of each cantilever are physically divided into two parts, which serve as a sensor and an actuator, respectively. The cantilevers are ring-shaped to improve the fill factor for a specific mirror size. An AC voltage with 180° phase difference can be applied to each pair of actuators *A*, *C* or *B*, *D*, in order to realize the deflection, and the mirror can make a 2D scan when all the actuators are driven simultaneously. 

**Figure 2:**
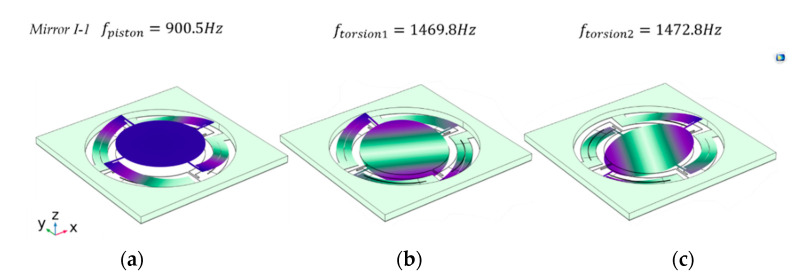
The mode shape results of *Design I*: (**a**) the first mode of the mirror is piston mode; in this mode, the mirror resonates along the z-axis; (**b**) the second mode is the tip mode and resonates at 1469.8 Hz; (**c**) the tilt mode resonates at 1472.8 Hz.

The structure was simulated using COMSOL, as shown in Figure 2. In this work, a 5 mm aperture mirror was used in *Design I* (*Mirror I*-*1*). Parametric scanning was executed to determine the optimized structure parameters, which are listed in Table 1. According to the mirror aperture and the chip size, the fill factor of *Mirror I*-*1* is 19.6%. The model used a substrate with a thickness of 400 μm Si, and the bottom surface of the substrate was fixed. A 30 μm device layer functioned as the movable parts of the device. The top and bottom electrodes were omitted since the thickness is usually sub-μm and the piezoelectric material was set to 1 μm. The resonance mode of the mirror was determined by modal analysis. The first resonance mode is piston mode, which was set at 900.5 Hz, and then the tip modes occur at around 1470 Hz. The difference between the piston mode and the first torsion mode is more than 500 Hz; this means the structure offers a good piston and torsion mode separation. The difference between the two torsion modes, which may arise because of the unsymmetrical mesh distribution of the FEM simulation, is less than 5 Hz, as exhibited in Figure 2. The coordinate system formed by tip and tilt axes, which are orthogonal to each other, rotates with the drive frequency.

### 2.2. Mirror with Gimbaled Structure (Design II)

**Figure 3:**
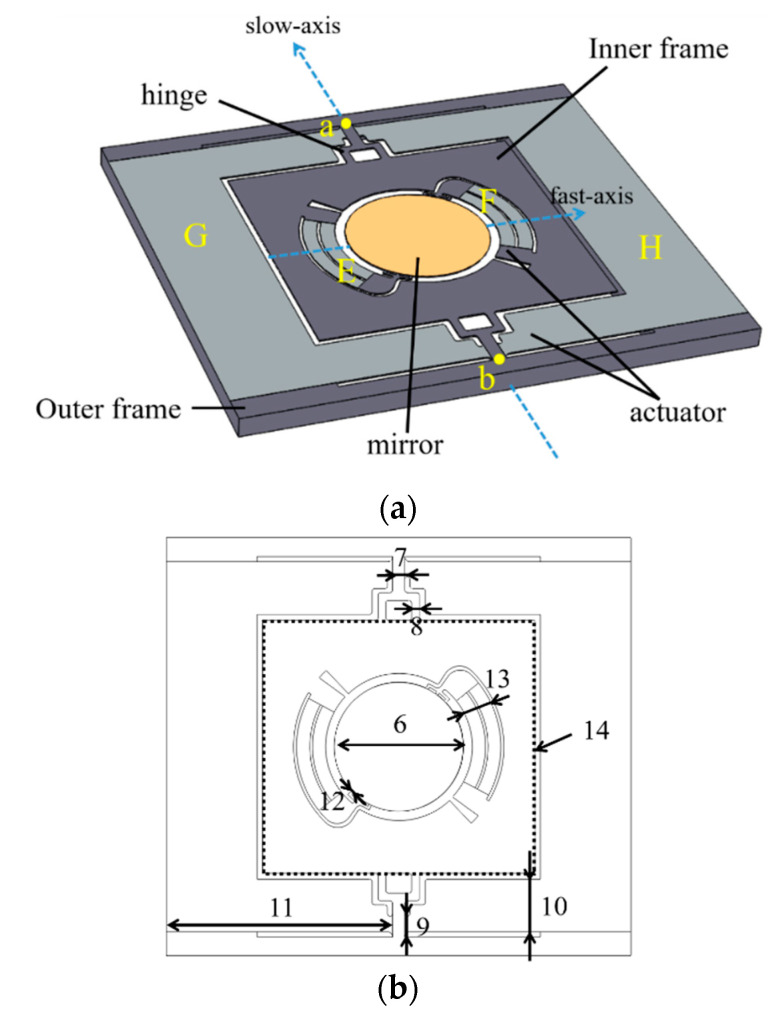
(**a**) The structure of *Design II*. (**b**) *Design II* parameter index.

Figure 3 depicts the geometry of *Design* *II*. Two ring-shaped cantilevers *E* and *F* are fixed on the inner frame and evenly distributed around the mirror. The far ends of the cantilevers connect the mirror with springs and actuate the mirror. The inner frame is made of the SOI wafer’s device layer and joins the outer frame at frame anchors, points *a* and *b*, as shown in the figure. The slow actuators *G* and *H* transfer the displacement to the inner frame through hinges. The hinges were designed to magnify the tip–tilt angle of the slow axis. The end point of the slow axis actuator transmits the maximum displacement to the hinge edge, resulting in a wide angle of the inner frame. The inner frame provides a counterweight balance to the rotation of the fast axis. When the mirror rotates arounds the fast axis, the inner frame remains essentially stationary. 

**Figure 4:**
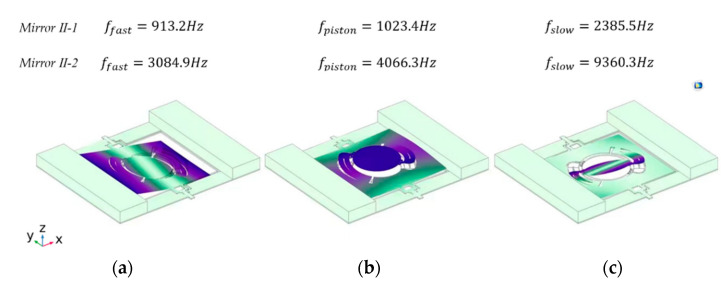
The mode shape results of *Design II*: (**a**) the first mode of the mirror is the torsion mode of the slow axis; (**b**) the second mode is piston mode, with the frame piston along the z-axis; (**c**) the fast axis resonates with high frequencies, which offers a significant difference between the two torsion modes.

The frequency analysis was also performed utilizing Finite Element Simulation. In this work, mirrors with 3 mm and 1.5 mm aperture were designed to compare the performance of different aperture micromirrors and are referred to as *Mirror II-1* and *Mirror II-2*, respectively, and the optimized structure parameters are listed in Table 2. The basic settings of the simulation are the same as in Section 2.1. *Mirror II-1* possesses a mirror diameter of 3 mm, a frame anchor width of 280 μm, and a chip size of 10.8 mm × 8.6 mm. According to the simulation results in Figure 4, in *Mirror II-1*, the first resonance frequency occurs at 913.2 Hz, where the inner frame including the mirror rotates along the slow axis. The piston mode frequency occurs at 1023.4 Hz, while the third resonance frequency, which occurs at 2385.5 Hz, represents the fast axis tilting mode. Meanwhile, the slow axis resonance frequency of *Mirror II-2* occurs at 3084.9 Hz, while the tilting mode of the fast axis resonates at 9360.3 Hz. The frequency difference between the two torsion modes is more than 2000 Hz for *Mirror II-1* and more than 6000 Hz for *Mirror II-2*. *Design II* thus offers a better torsion mode separation compared to *Design I*. The simulation results of the mirrors demonstrate that eliminating the mechanical axis-coupling results in high performance. Additionally, the fill factors of these two mirrors were calculated to be 7.6% and 4.8%, which are significantly smaller than those in *Design I*.

## 3. Fabrication Process

**Figure 5:**
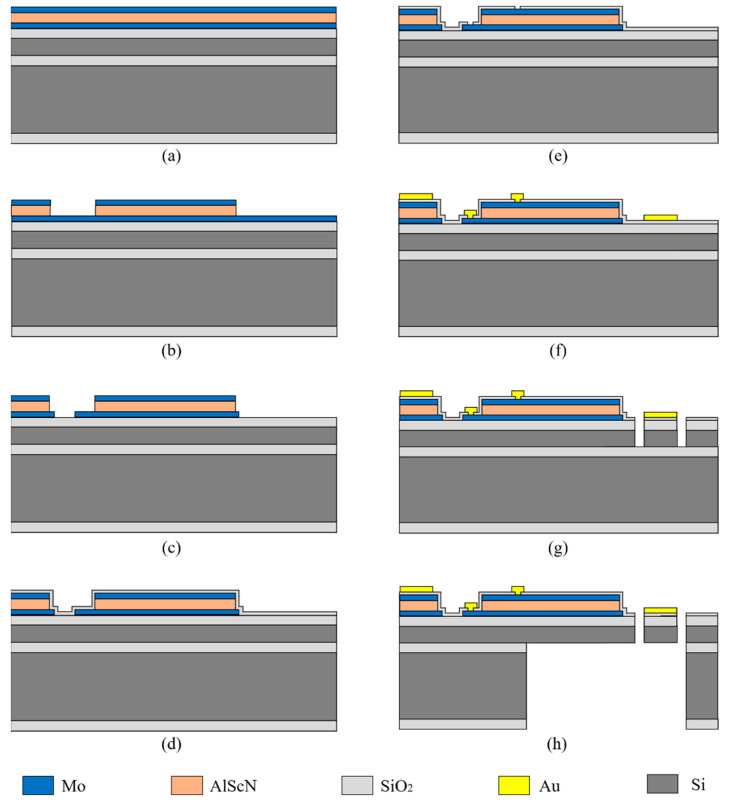
Fabrication process flow. (**a**) Deposition of the bottom electrode Mo, the piezoelectric layer AlScN, and the top electrode Mo on LPCVD SiO_2_; (**b**) Patterning the top electrode Mo and wet-etching AlScN using 25% TMAH at room temperature; (**c**) Patterning the bottom electrode Mo; (**d**) Depositing PECVD SiO_2_ on the top Mo; (**e**) Dry-etching PECVD SiO_2_ by RIE; (**f**) Sputtering and etching of Ti and Au; (**g**) Etching the device layer through DRIE; (**h**) Patterning the handle layer to release the device.

**Figure 6:**
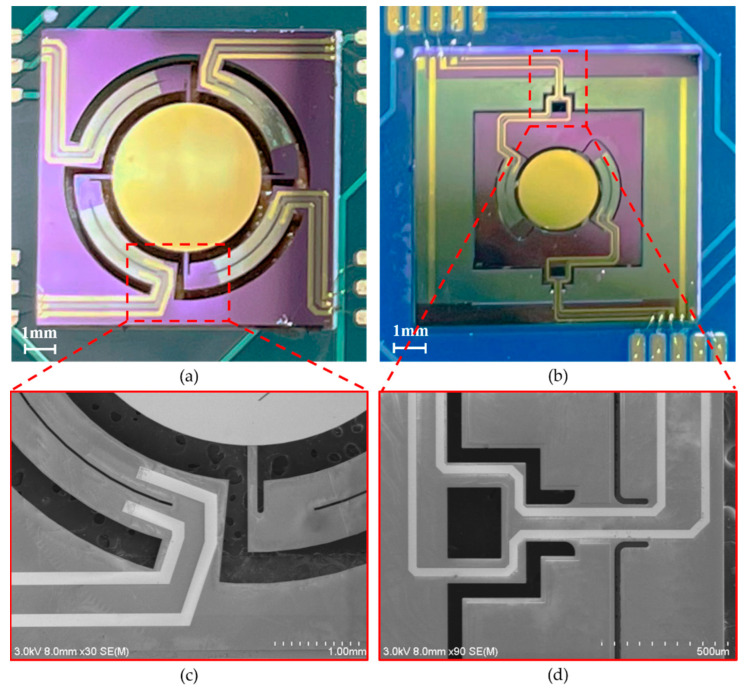
(**a**) Photograph of *Design I*. (**b**) Photograph of *Design II*. (**c**) SEM of *Design I* spring. (**d**) SEM of *Design II* hinge.

The aforementioned mirrors were fabricated on the same wafer to avoid the wafer-to-wafer nonuniformity. An SOI wafer with a 400 μm handle layer, a 2 μm buried oxide layer, and a 30 μm device layer was utilized as the starting material. A 100 nm LPCVD (low-pressure chemical vapor deposition) silicon oxide was deposited on top of the device layer serving as the electrical insulation between the bottom electrode and Si. Then, the device was fabricated following the steps in Figure 5: (a) the bottom electrode Mo (200 nm), the piezoelectric layer AlScN (1 μm), and the top electrode Mo (200 nm) were magnetron-sputtered in sequence on LPCVD SiO_2_; (b) the top electrode Mo was etched by an ion beam using patterned photoresist as mask; the AlScN layer was wet-etched using 25% TMAH (Tetramethylammonium hydroxide) at room temperature, where the top electrode Mo served as hard mask; (c) the pattern of bottom electrode Mo was transferred from photoresist via ion beam etching; (d) a 200 nm silicon oxide was deposited on the top Mo in a PECVD (Plasma-Enhanced Chemical Vapor Deposition) process; (e) it was dry-etched by RIE; (f) then, 30 nm Ti and 200 nm Au was sputtered on the wafer and pattered to form a mirror surface and a wire bonding pad; (g) the hinge, frame, actuator, and mirror were profiled by removing certain parts of Si from the device layer through DRIE (Deep Reactive Ion Etching); (h) finally, the wafer was trenched from the back side to release all the movable parts of the device. The DRIE etching step automatically stopped at the buried oxide layer, and BOE was used to remove the remaining buried oxide layer. The photographs and SEM of fabricated mirrors are shown in Figure 6. 

## 4. Results and Discussion

### 4.1. Frequency Response

**Figure 7:**
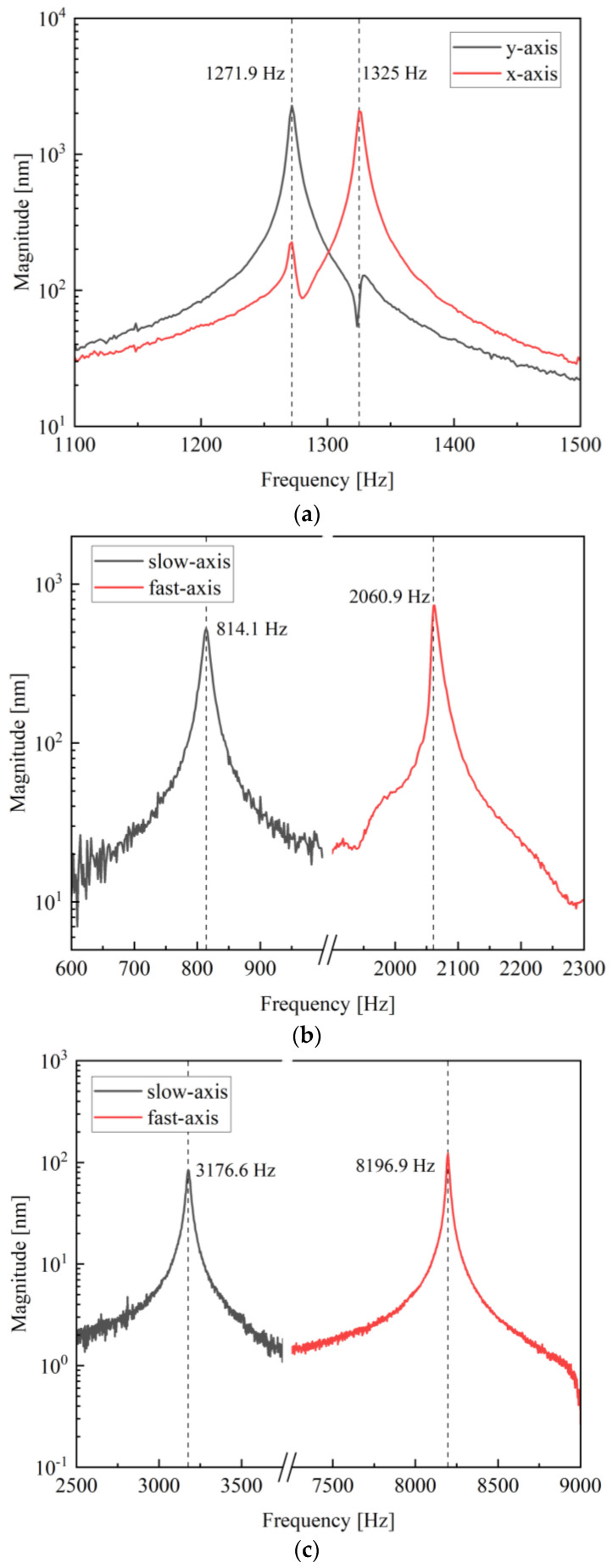
Resonance spectrum of (**a**) *Mirror I-1*, (**b**) *Mirror II-1* and (**c**) *Mirror II-2* for two perpendicular axes.

Firstly, the frequency responses of the proposed mirrors were characterized using a Laser Doppler Velocimeter (Polytec MSA 500). Figure 7 indicates the single-axis frequency responses of *Mirror I-1*, *Mirror II-1* and *Mirror II-2*. Since the structure of *Mirror I-1* follows *Design I*, the x-axis and y-axis of the mirror were central symmetric, and the resonant frequencies of the two axes were similar, as revealed in Figure 7a. The red curve represents the x-axis frequency response of *Mirror I-1* and the black curve represents the y-axis resonant frequency. The frequency response of the x-axis was located at 1325.0 Hz with a Q factor of 318, and the frequency response of y-axis appeared at 1271.9 Hz with a Q factor of 265. The frequency difference between the two axes was less than 50 Hz, which is higher than the simulation result because of the fabrication tolerance and the mismatch of capacity between actuators and driving circuits. First, the thickness and stress of the deposited films, such as PECVD SiO2 and Mo/AlScN/Mo, influenced the stiffness of the actuator, so the uniformity of the film thickness during fabrication should be optimized, and the stress between different films should be matched. Second, as the shape of the actuator was mainly determined by the first DRIE process from the front side of the wafer, and the actuator was released by the second DRIE process from the back side, the alignment accuracy between these two processes was also a dominant factor that influenced the mismatch between the resonant frequencies of the two axes. *Mirror II-1* and *Mirror II-2* took the geometry of *Design II*, and both had sperate resonant frequencies for the fast and slow axes. As shown in Figure 7b, the slow axis of *Mirror II-1* resonated at 814.1 Hz when the fast axis reached its highest amplitude at 2060.9 Hz. The frequency difference of *Mirror II-1* between the two axes was more than 1000 Hz, and the Q factors of the slow and fast axes were 74 and 217, respectively. Likewise, *Mirror II-2* had two main resonant frequencies at 3176.6 Hz (Q factor = 132) and 8196.9 Hz (Q factor = 546) for the vertical and horizontal axes, respectively. 

### 4.2. Deflection Characteristics in Atmosphere

**Figure 8:**
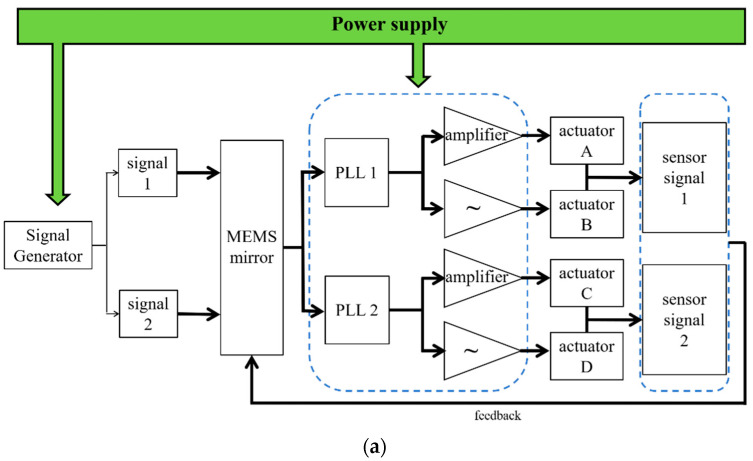

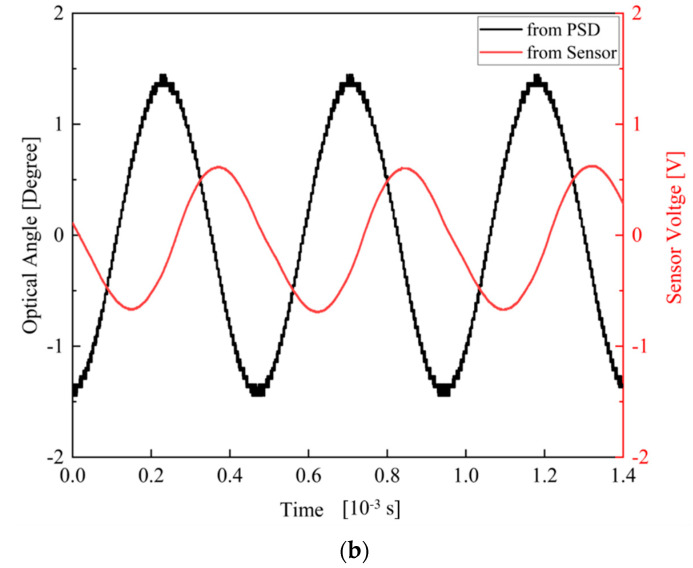
(**a**) Architecture of the closed-loop driving system for the MEMS mirrors. (**b**) Waveform of the angle signal of *Mirror I-1*; PSD signal in black; sensor signal in red.

To meet the purpose of 2D scanning, the mirrors needed to be tilted by actuators to achieve biaxial scanning. As illustrated in Figure 8a, the signal generator generated a sine signal with the desired driving frequency. The signals passed through a Phase-Locked Loop (PLL) before the signals were amplified and inverting amplified, respectively, so that the two actuators for the x-axis could be driven with 180° phase difference. These two signals drove the two actuators of the same axis, around which the mirror rotated and deflected the laser. As mentioned in Section 2, each cantilever of *Design I* and the fast axis of *Design II* possesses both actuator and sensor parts, which was achieved by physically separating the piezoelectrical films and the device silicon. When the cantilevers resonate at driving frequency, the sensors will generate a voltage signal with the same frequency due to the positive piezoelectric effect. The sensor signal will be feedbacked to the MEMS mirror to realize the closed-loop control. To reflect the real-time position information of the micromirror, a Position-Sensitive Detector (PSD) was placed on the optical path. When the micromirror deflects, the PSD detects the movement of the laser spot and converts this information into a voltage signal output. Figure 8b represents the sensor signal (the red line) detected during the scanning; the black line is the optical angle of the micromirror converted from the PSD voltage signal. Obviously, the signal from the sensor and the deflecting of the micromirror are of the same frequency, and the phase difference between them may have come from the position difference of the actuator and the sensor on the device, as well as from the driver circuit. When the mirror worked in torsion mode, the deformation of the inner and outer ring piezoelectric films varied depending on the radius at which they were located. For a mirror’s resonant system, the phase response of the cantilever is π/2 [32]. Furthermore, the phase difference exits in the control circuit were also reflected in the figure since the sensor signal was detected by the circuit. This phase difference between the actuator and the sensor may be compensated through the circuit, so that the position of the mirror can be correctly detected.

**Figure 9:**
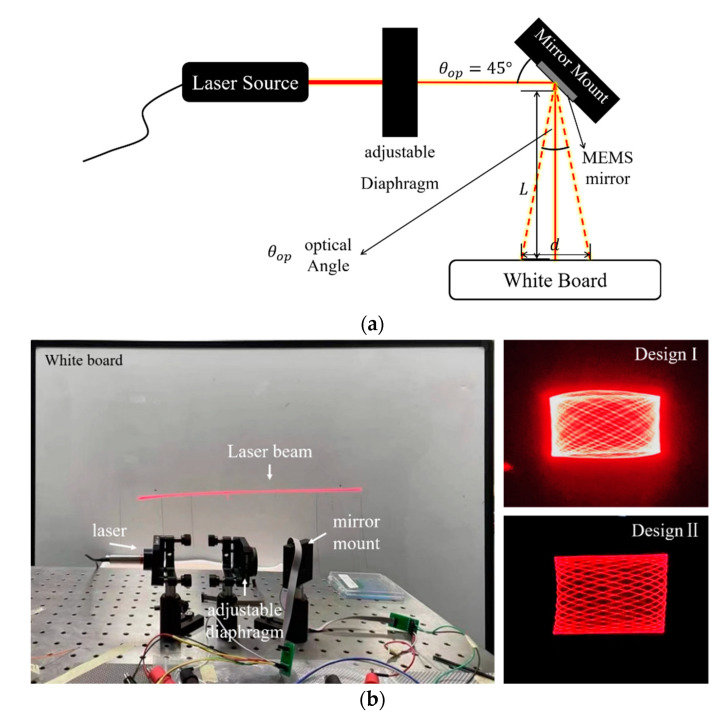
(**a**) Schematic diagram of the scanning platform. (**b**) The built platform and the scanning results of *Design I* and *Design II*.

**Figure 10:**
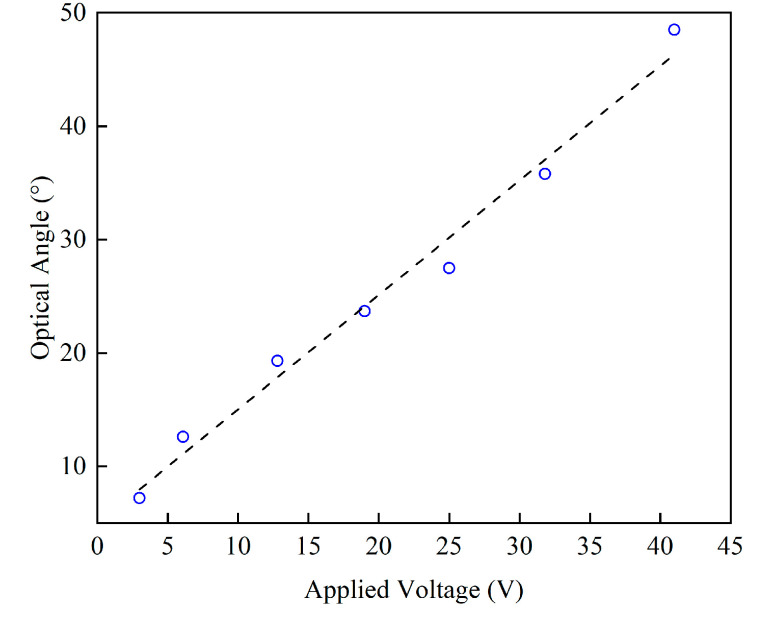
The dependence of the optical angle on the applied voltage for the fast axis of *Mirror II-1*.

**Figure 11:**
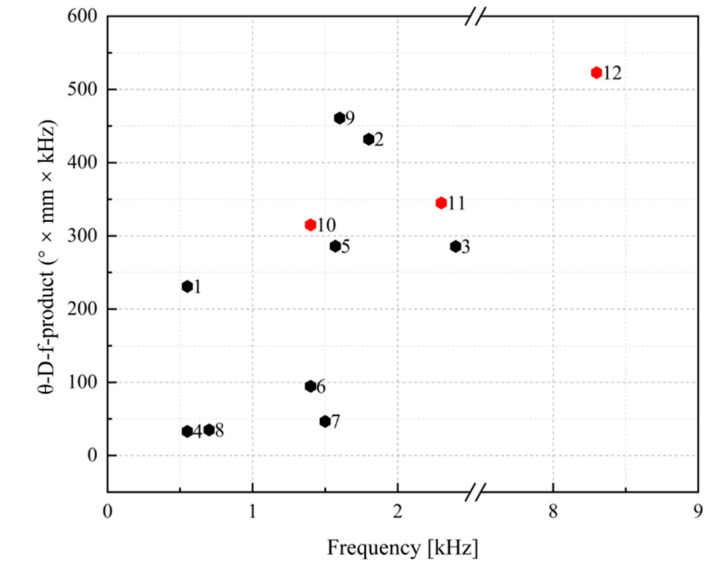
Comparison of MEMS mirrors for LiDAR application. *Mirror I-1*, *Mirror II-1* and *Mirror II-2* are represented by 10, 11 and 12, respectively, in this work. 1 = [12], 2 = [29], 3 = [33], 4 = [34], 5 = [35], 6 = [36], 7 = [37], 8 = [38], 9 = [39], 10 = *Mirror I-1*, 11 = *Mirror II-1*, 12 = *Mirror II-2*.

A 2D scanning platform was also set up. Figure 9a illustrates the laser scanning platform layout. The laser was collimated to the MEMS mirror through an adjustable diaphragm with an angle of 45°. This was to ensure the perpendicularity between the laser and the projection screening. The optical angle of the tested mirror was defined using the following equation: $\theta_{op}=2\times \arctan\left(\frac{L}{2d}\right)$, where L is the length of the scanning beam and d is the distance between the center of the mirror and the projection plane. As shown in (b), the platform included a laser source, a mirror mount, an adjustable diaphragm, and a white board serving as the projection plane. When the x- and y- axes were driven synchronously at their own resonant frequencies, respectively, a Lissajous scanning could be realized. The biaxial scanning of *Mirror I-1* is displayed on the top right. The laser beam overlaps on the edge of the scanning area because of the mechanical coupling of *Design I*. By comparison, the Lissajous patterns of *Design II* (bottom right) were rectangular. This verifies the mechanical decoupling of the gimbaled structure. Three biaxial scanned laser beams at 30 V on different mirrors are exhibited in Table 3 for comparison. The maximum optical angle of *Mirror I-1* reached only 11.4° × 8.6°. The scanning range of *Mirror II-1* was 29.4 cm × 3.3 cm when the projecting distances were equal to 50 cm, corresponding to 32.8° × 3.8° for the optical tilting angle, while *Mirror II-2* delivered an optical tilting angle of 22.6° × 4.1° under the same condition. The fast axis of *Mirror II-1* had the highest FOV-D-product of 98.4 [° × mm], while the FOV-D-products of *Mirror II-2* reached only 33.9 [° × mm]. Higher voltages were also applied to characterize the maximum deflection of the mirrors: *Mirror II-1* reached 48.5° total optical angle at 41 V, while *Mirror I-1* and *Mirror II-2* reached 45°and 42°, respectively. The dependence of the optical angle with an applied voltage is verified to be in direct proportion, as shown in Figure 10. Accordingly, a comparison between the MEMS mirrors for LiDAR application presented in this work and those presented in other published work on this topic is given in Figure 11. There, it can be seen that *Mirror II-2* has the advantage of possessing a large resonance frequency and a wide tilting angle. However, for different aperture mirrors of the same design, the influence of the mirror size on the FOV-D product cannot be ignored since the optical angle of *Mirror II-1* is larger than that of *Mirror II-2*.

### 4.3. Deflection Characteristics in Vacuum 

**Figure 12:**
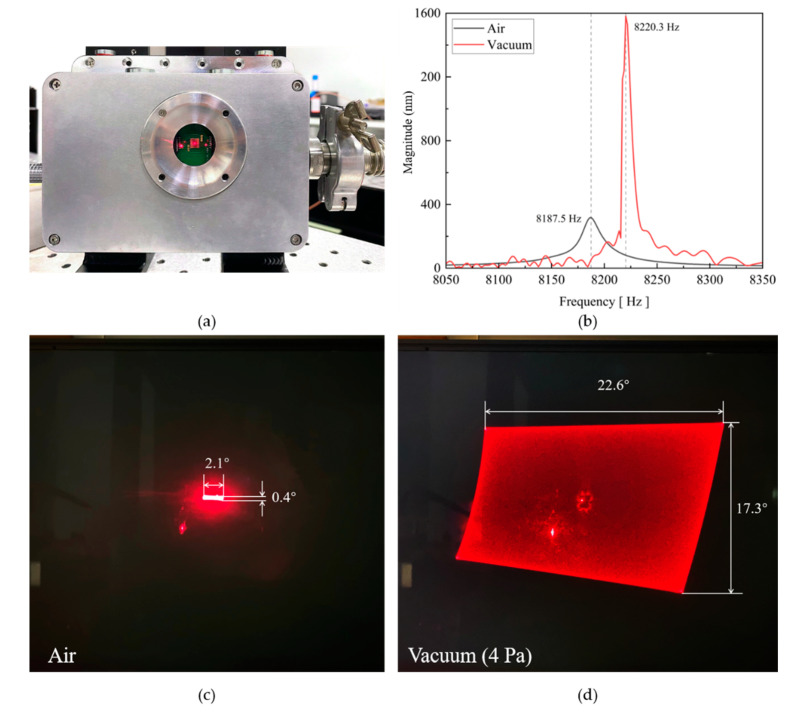
(**a**) Vacuum chamber with glass viewport. (**b**) Frequency responses of *Mirror II-2* in air (black line) and vacuum (red line). (**c**) Biaxial scanned laser beams of *Mirror II-2* at atmosphere and (**d**) under vacuum condition (4 Pa).

To further study the characteristic of the gimbaled structure mirror in vacuum conditions, *Mirror II-2* was mounted in a vacuum chamber (Figure 12a) with glass viewport, through which a laser was pointed at the mirror. First, the frequency responses of *Mirror II-2* in air and in a vacuum are shown in Figure 12b. There, it can be seen that the resonance frequency shifts from 8187.5 Hz (air) to 8220.3 Hz (vacuum). Then, the fast axis was actuated at 1 V and the slow axis at 10 V. The results at atmosphere in Figure 12c show that the FOV reaches its maximum at 8187.1 Hz × 3187.7 Hz by only 2.1° × 0.4° (fast axis × slow axis). Then, the vacuum chamber was pumped to 4 Pa. The performed projection is shown in Figure 12d. The tilting angle in the vacuum chamber was 22.6° × 17.3°, which is more than 10 times larger than the one at atmosphere. In addition, both the projection patterns at atmosphere and in air had a red point in the middle. This was caused by direct reflection of the incoming laser beam at the glass cover of the vacuum chamber, which could be eliminated by a tilt glass window [40]. Therefore, a vacuum package with a tilted window is preferred for large FOV applications. It is also obvious that the scanning area of the mirror is not perfectly rectangular. This is because the incident angle of the laser is 45°, which causes a bending of the scanned laser. One way to correct the images is to add a beamsplitter between the laser and the micromirror to ensure the vertical incidence of the laser. Another method is to calculate the amount of graphic distortion, based on the scanning angle, so that the scanned image can be corrected by an algorithm. 

## 5. Conclusions

In summary, three types of piezoelectrical MEMS mirrors are presented in this article, with AlScN film used as the functional layer. The mirrors were fabricated in a MEMS process using SOI wafers as the substrate. By integrating a piezoelectric sensor, position information was extracted from the sensor voltage during the operation of the micromirrors to achieve closed-loop control. The LDV frequency characteristic results obtained from these three mirrors validate the conclusion that non-gimbaled micromirrors are plagued by mechanical coupling, while the mechanical coupling phenomenon of gimbaled micromirrors is virtually negligible. The fast axis optical tilting angle of 3 mm gimbal-less mirror reached 48.5° at a driving voltage of 41 V, which was the highest among the designed mirrors. Mirrors with smaller apertures turn out to have a wider optical tilting angle under the same voltage, which leads to a trade-off between large mirror size and wide FOV in LiDAR application. Furthermore, a vacuum scanning was performed to demonstrate the necessity of vacuum packaging of mirrors for wide FOV application, since the optical angle was magnified by more than 10 times in a vacuum (4 Pa) and reached 22.6° with a mere 1 V driving voltage. 

## Figures and Tables

**Table 1:** The main parameters of *Mirror I-1*.

Index	*Design I* Parameter	*Mirror I-1*	Units
1	mirror diameter	5	mm
2	spring notch length	400	μm
3	spring width	100	μm
4	cantilever width	850	μm
5	cantilever root length	400	μm
chip size		10 × 10	mm × mm

**Table 2:** The main parameters of *Mirror II-1* and *Mirror II-2*.

Index	*Design II* Parameter	*Mirror II-1*	*Mirror II-2*	Units
6	mirror diameter	3	1.5	mm
7	frame anchor width	280	140	μm
8	hinge width	180	90	μm
9	slow axis anchor width	540	270	μm
10	slow axis width	1.22	0.61	mm
11	slow axis length	3.16	1.58	mm
12	spring width	60	30	μm
13	cantilever width	680	340	μm
14	frame size	3.17 × 2.94	1.54 × 1.47	mm× mm
chip size		10.8 × 8.6	5.4 × 4.3	mm× mm

**Table 3:** Biaxial scanned laser beam characteristics.

Design	Mirror	Optical Angle [°]	Applied Voltage [V]	FOV-D Product [° × mm]
*I*	*Mirror I-1*	11.4 × 8.6	30	57
*II*	*Mirror II* *-1*	32.8 × 3.8	30	98.4
	*Mirror II* *-2*	22.6 × 4.1	30	33.9

## Data Availability

Data are available from the authors on request.
